# 

**Published:** 1787

**Authors:** 


### THE LONDON MEDICAL JOURNAL.

### VOLUME THE EIGHTH.

FOR THE YEAR 1787.

S.G.O. LIBRARY

Surgeon Gen'l's Office LIBRARY. 63447 Washington, D.C.

LONDON:

PRINTED for the EDITOR,

And ſold by JOSEPH JOHNSON, LONDON;

CHARLES ELLIOT, EDINBURGH;

And P. BYRNE, DUBLIN.

M,DCC,LXXXVII.

63447

### CATALOGUE OF BOOKS.

1. OBSERVATIONS in Midwifery, particularly on the different Methods of affifting Women in tedious and difficult Labours; to which are added, Obfervations on the principal Diforders incident to Women and Children. By *William Deafe*, furgeon to the united hofpitals of St. Nicholas and St. Catharine. 8vo. Dublin, 1783.

2. The chemical Effays of *Charles William Scheele*, tranflated from the Tranfactions of the Academy of Sciences at Stockholm, with Additions. 8vo. *Murray*, London, 1786.

3. The Anatomy of the abforbent Veffels of the human Body. By *William Cruikfhank*. 4to. *Nicol*, London, 1786.

4. Clinical Obfervations on the Ufe of Opium in low Fevers, and in the Synochus; illuftrated by Cafes, Remarks, &c. By *Martin Wall*, M. D. Lord Litchfield's clinical Profeffor, one of the Phyficians to the Radcliffe Infirmary, and late Fellow of New College, Oxford. 8vo. *Cadell*, London, 1786.

5. Obfervations upon the new Opinions of John Hunter, in his late Treatife on the venereal Difeafe. By *Jeſſe Foot*, Surgeon. 8vo. Parts I. and II. *Becket*, London, 1786.

6. A practical Treatife on the Prevention and Cure of Difeafes in general. By Dr. *John Memis*, Phyfician in Aberdeen, and a Manager of the Royal Infirmary in that City. 8vo. Aberdeen, 1786.

7. A Maritime State confidered, as to the Health of Seamen; with effectual Means for rendering the Situation of that valuable Clafs of People more comfortable. To which are annexed, fome general Obfervations on the Difeafes incident to Seamen; and an Appendix of additional Notes and Remarks in the order of the Work. By *Charles Fletcher*, M. D. late Surgeon in His Majefty's Navy. 8vo. Dublin, 1786.

8. An Inquiry into the prefent State of medical Surgery, Vol. II. By *Thomas Kirkland*, M. D. Member of the Royal Medical Society at Edinburgh. 8vo. *Dawfon*, London, 1786.

9. A Reply to Dr. Berkenhout's Dedication to each individual Apothecary in England, (prefixed to his Symptomatology.) By Somebody who is a Lover of Candour. 8vo. *Rivington*, London, 1786.

10. Obfervations on certain Parts of the Animal mal Oeconomy. By *John Hunter*. 4to. London, 1787.

11. Short Directions for the Management of Infants. By *T. Mantell*, Surgeon, and Practitioner in Midwifery at Dover. 12mo. *Becket*, London, 1787.

12. An Account of the Effects of Swinging, employed as a Remedy in Pulmonary Consumption and Hectic Fever. By *James-Carmichael Smyth*, M. D. F. R. S. Physician Extraordinary to His Majesty. 8vo. *Johnson*, London, 1787.

13. *A. Jos. Testa*, Phil. et Med. D. in magno Ferrariensium Nosocomio, Med. et Chir. Prof. ord. De vitalibus periodis Ægrotantium et Sanorum: seu Elementa Dynamicæ Animalis. 8vo. 2 Vol. *Johnson*, London, 1787.

14. Tentamen Medicum Inaug. de Variolis inserendis. Auctore *Georgio Bachmetiev*, Mosc oviense. 8vo. Edin. 1786.

15. Dissertatio Medica Inaug. de Phrenitide idiopathica. Auctore *Thoma Burnside*, Hiberno. 8vo. Edin. 1786.

16. Dissertatio Medica Inaug. de Rheumatismo acuto. Auctore *Hugone Casement*, Hiberno. 8vo. Edin. 1786.

17. Dissertatio Medica Inaug. quædam de

 Morbo

Morbo Venereo complectens. Auctore *Nicolao Elcock*, Hiberno. 8vo. Edin. 1786.

18. Disputatio Medica Inaug. quædam de Vestitu Laneo tradens. Auctore *Johanne H. Gibbons*, Pennsylvaniense. 8vo. Edin. 1786.

19. Dissertatio Physica Inaug. de Igne. Auctore *Hugone Gillan*, Scoto. 8vo. Edin. 1786.

20. Dissertatio Medica Inaug. de Morbo Morteque submersorum investigandis. Auctore *Edmundo Goodwyn*, Anglo. 8vo. Edin. 1786.

21. Tentamen Medicum Inaug. quædam de Placenta proponens. Auctore *Jacobo Jeffray*, Scoto. 8vo. Edin. 1786.

22. Dissertatio Chemica Inaug. de duabus Aeris speciebus Aquam gignentibus. Auctore *Georgio Kirkaldie*, Scoto. 8vo. Edin. 1786.

23. Tentamen Medicum Inaug. de reciproca atque mutua Systematis sanguinei et nervosi Actione. Auctore *Ignatio Maria Ruiz Luzuriaga*, Cantaber-Hispano. 8vo. Edin. 1786.

24. Disputatio Physiologica Inauguralis circa novi Genituram Animalis. Auctore *Samuele Latham Mitchill*, de Civitate Novo-Eboraco. 8vo. Edin. 1786.

25. Dissertatio Medica Inaug. de Colica Pictonum. Auctore *Joanne Prendergast*, Hiberno. 8vo. Edin. 1786.

26. Spi-

26. Spicilegium Medicum Inaug. de Morbillis. Auctore *Jacobo Watson Roberts*, ex insula Antigua. 8vo. Edin. 1786.

27. Tentamen Medicum Inaug. de Rubeola. Auctore *Gulielmo Whitelaw*, Hiberno. 8vo. Edin. 1786.

28. *Samuelis Foart Simmons*, Medici Londinensis, Observationes practicæ de Phthisi Pulmonali, quas ex Anglico idiomate in Latinum vertit *F. A. Van Zandycke*, M. D. 8vo. Brugis Flandrorum, 1786.

29. Observationes Medicinales de febribus intermittentibus & qua ratione eisdem Medendum sit. Opus, quod Scientiarum Artium atque Literarum Academia Divionensis præmio coronavit die 11 Augusti, 1782. Auctore *Carolo Strack*, M. D. & in Universitate Moguntina Praxeos Medicæ & Collegii Clinici Professore, p. o. Eminentiss. ac Celsiss. Princip. Elector. Moguntini Consil. Aulic. &c. 8vo. Offenbach, 1785.

30. *Wenceslai Trnka de Krzowitz*, S. R. I. Equitis, Medicinæ Doct. in Reg. Universit. Pestiensi Pathol. Prof. p. o. Historia Cardialgiæ, omnis Ævi Observata Medica Continens. 8vo. Vindobonæ, 1785.

31. Des Maladies des Filles; par M. *Chambon de Montaux*, Medecin de la Faculté de Paris, de la Société Royale de Medecine. Pour servir de suite aux Maladies des Femmes du même Auteur. 8vo. Paris, 1785, 2 vol.

32. Des Maladies de la Grossesse; par M. *Chambon de Montaux*. Pour completter l'Histoire des Maladies des Femmes du même Auteur. 8vo. Paris, 1785, 2 vol.

33. Observations sur les Maladies Veneriennes, par feu M. *A. Nunes Ribeiro Sanchés*; publiées par M. *Andry*. 12mo. Paris, 1785.

34. Methode de traiter les morsures des Animaux enragés, & de la Vipere; suivie d'un precis sur la Pustule maligne. Par M. *Enaux*, Professeur du Cours d'Accouchements des Etats de Bourgogne; et par M. *Chaussier*, Professeur d'Anatomie des Etats de Bourgogne, &c. 8vo. Dijon, 1785.

35. Extrait des Registres de l'Academie Royale des Sciences, du 22 Novembre, 1786. Rapport des Commissaires chargés, par l'Academie, de l'examen du Projet d'un nouvel Hotel Dieu. 4to. Paris, 1786.

36. D. *William Saunders* Beobachtungen ueber die vorzueglichen heilkraefte der rothen Peruvianischen Rinde. Aus dem Englischen, nach nach der dritten Aufgabe. 8vo. Leipsic, 1784.

37. Abhandlung von dem nutzen der Spanischen Fliegenpflaster in soporœsen Wechselfiebern, *i. e.* Essay on the Use of Blisters in soporose intermitting Fevers. By *H. Wolff*, M. D. Physician at Altona. 8vo. Altona, 1785.

38. Magazin fuer Apotheker, Chymisten und Materialisten. *i. e.* Magazine for Apothecaries, Chemists, and Druggists. By *J. Carp. Philip Elwert*. 8vo. Nurenberg, 1785.

39. Saturnus redivivus; eine neue Betrachtung ueber die Bleymittel, besonders ueber das Bleyextrakt; von einem Feldwundaerzte der K. K. Oesterreischen Armee. *i. e.* Saturnus redivivus; a new Treatise on Lead, particularly on the Extract of Lead. By a Surgeon of the Austrian Army. 8vo. Vienna, 1785.

40. Abhandlungen der Schwedischen Aerzte, oder Sammlung seltener Beobachtungen und Faelle, aus allen theilen der Medicin, vorzueglich aber aus der Praktischen Arzneywissenschaft und Chirurgie. *i. e.* Essays of the Swedish Physicians; or a Collection of rare Observations and Cases, in all Parts of Physic, but principally in the Practice of Physic and Surgery.

gery. Tranflated from the Latin by *J. J. Roemer*. Vol. I. 8vo. St. Gall, 1785.

41. Tafchenbuch fuer Deutfche Wundaerzte. *i. e.* A Manual for German Surgeons, for the Years 1784 and 1785. 8vo. Altenburg, 1785.

42. Hippokrates Werke; aus dem Griechifchen ueberfetzt, und mit Erlæuterungen. *i. e.* The Works of Hippocrates, tranflated from the Greek, with explanatory Notes. By *John Frederick Charles Grimm*, M. D. Aulic Counfellor aud Body Phyfician to the reigning Duke of Saxe Gotha. Vol. II. 8vo. Altenburg, 1784.

43. Abhandlung von dem Krebs und der beften Heilart deffelben. *i. e.* [A Treatife on Cancer, and the beft Method of treating it. By *J. H. Jaenifch*, M. D. 8vo. 2d edition. Peterfburgh, 1785.

44. De Ruptura Cordis prefide *Adolpho Murray* pro gradu Doctoris differit *Petrus Guftav. Tengmalm*. 4to. Upfaliæ, 1785.

45. Differtatio Chirurgica de tumoribus falivalibus; quam præfide *Adolpho Murray* pro gradu Doctoris defendit *Joannes Guftavus Lodin*. 4to. Upfaliæ, 1785.

46. *Joannis Jacobi Hartenkeil*, M. D. Tractatus de Veficæ Urinariæ Calculo. 4to. Bambergæ, 1785. c. Tab. Æn. iv.

